# Stereotactic biopsy for lesions in brainstem and deep brain: a single-center experience of 72 cases

**DOI:** 10.1590/1414-431X2021e11335

**Published:** 2021-07-23

**Authors:** Feng Qin, Zhenchao Huang, Qing Dong, Xiaofeng Xu, Tingting Lu, Jianning Chen, Na Cheng, Wei Qiu, Zhengqi Lu

**Affiliations:** 1 Department of Neurosurgery, the Third Affiliated Hospital of Sun Yat-Sen University, Guangzhou, Guangdong, China; 2 Department of Neurology, the Third Affiliated Hospital of Sun Yat-Sen University, Guangzhou, Guangdong, China; 3 Department of Pathology, the Third Affiliated Hospital of Sun Yat-Sen University, Guangzhou, Guangdong, China

**Keywords:** Stereotactic brain biopsy, Deep brain, Brainstem, Internal capsule, Extraventricular approach

## Abstract

Stereotactic biopsies for lesions in the brainstem and deep brain are rare. This study aimed to summarize our 6-year experience in the accurate diagnosis of lesions in the brain stem and deep brain and to discuss the technical note and strategies. From December 2011 to January 2018, 72 cases of intracranial lesions in the brainstem or deep in the lobes undergoing stereotactic biopsy were retrospectively reviewed. An individualized puncture path was designed based on the lesion's location and the image characteristics. The most common biopsy targets were deep in the lobes (43 cases, 59.7%), including frontal lobe (33 cases, 45.8%), temporal lobe (4 cases, 5.6%), parietal lobe (3 cases, 4.2%), and occipital lobe (3 cases, 4.2 %). There were 12 cases (16.7%) of the brainstem, including 8 cases (11.1%) of midbrain, and 4 cases (5.6%) of pons or brachium pontis. Other targets included internal capsule (2 cases, 2.8%), thalamus (3 cases, 4.2%), and basal ganglion (12 cases, 16.7%). As for complications, one patient developed acute intracerebral hemorrhage in the biopsy area at 2 h post-operation, and one patient had delayed intracerebral hemorrhage at 7 days post-operation. The remaining patients recovered well after surgery. There was no surgery-related death. The CT-MRI-guided stereotactic biopsy of lesions in the brainstem or deep in the brain has the advantages of high safety, accurate diagnosis, and low incidence of complications. It plays a crucial role in the diagnosis of atypical, microscopic, diffuse, multiple, and refractory lesions.

## Introduction

The diagnosis of lesions in the brainstem and deep brain is still challenging. Neuroimaging, such as computed tomography (CT), magnetic resonance imaging (MRI) enhanced scan, and positron emission tomography (PET)-CT/MRI, has provided multiple levels of information regarding anatomy, metabolism, and neurological function, so that most common intracranial diseases can be effectively diagnosed according to neuroimaging and clinical diagnosis (1-4). However, in the diagnosis of atypical, small, multiple, diffuse lesions in the brain, the consistency rate among clinical, imaging, and pathological diagnoses is not high (5,6). Rachinger et al. (7) compared the diagnostic consistency of brainstem lesions between MRI imaging and pathological finding after stereotactic biopsy and found that the difference rate between the two diagnoses was as high as 30.4%. In the non-enhancing intracranial lesions, the consistency rate between MRI diagnosis and pathological diagnosis is only 26.1 to 48.9% (8,9). These results suggest that imaging diagnosis of intracranial lesions may lead to a considerable proportion of misdiagnosis, and therefore it is important to obtain a histopathological diagnosis.

Biopsy of intracranial lesions can be performed by craniotomy (10), neuronavigation (11), neuroendoscopy (12), and stereotactic biopsy (13). Image-guided stereotactic brain biopsy is a highly accurate, minimally invasive method to obtain the histological diagnosis of cerebral lesions. The CT/MRI-guided stereotactic brain biopsy has an error of <0.7 mm for locating lesions in the brain (14). Gessler et al. (10) have demonstrated that stereotactic brain biopsy can provide tumor molecular markers as accurately as open craniotomy. At present, most stereotactic biopsies are performed on relatively large lesions or those with relatively clear images. Studies on stereotactic biopsies for atypical lesions, tiny lesions, or those in the brainstem and the most important functional area in the deep brain are rare. From December 2011 to January 2018, we performed CT-MRI-guided stereotactic brain biopsy for 72 cases of intracranial lesions in the deep brain and brainstem in our hospital. The purpose of this study was to summarize our experience in these 72 cases and to discuss the technical note and strategies for stereotactic brain biopsy.

## Material and Methods

### Patients

From December 2011 to January 2018, 76 consecutive difficult cases of intracranial lesions in the brainstem or deep in the lobes undergoing stereotactic biopsy were retrospectively reviewed, and 72 of them that received CT-MRI-guided stereotactic biopsy were included. Patients meeting at least one of the following criteria were included: 1) unknown diagnosis and identified as difficult cases by multidisciplinary team discussion; 2) lesions located in the brainstem or deep brain (depth >3 cm); 3) biopsy results were critical for making treatment decisions, such as differential diagnosis of suspicious tumors, inflammation, degenerative lesions, postoperative recurrence of tumor, or necrosis; 4) patient intolerant to open craniotomy; and 5) lesions were small, multiple or diffuse, and difficult to be removed by craniotomy. Patients with surgical contraindications, such as vascular lesions, coagulopathy, local and/or systemic infections were excluded. This study was approved by the Institutional Review Board of the Third Affiliated Hospital of Sun Yat-Sen University and written informed consent was waived by the Board due to the retrospective nature of this study. Of the 72 cases in this study, partial data of 29 cases have been reported in a case series published in a Chinese journal (15), including 12 cases of midbrain or pons, 2 cases of internal capsule, 3 cases of thalamus, and 12 cases of basal ganglia. Also, partial data of the 2 cases of germinomas in this study have been reported in a case series (16).

### MRI and CT scans

All patients underwent preoperative contrast-enhanced T1- and T2-weighted MRI scans (Achieva 1.5T MRI system, Philips, USA) and contrast-enhanced CT scans (Aquilion One Genesis CT Scanner, Toshiba, Japan). Field of view (FOV)=280 mm. Scanning direction: from bottom to top; slice increment=1 mm, slice thickness=0.5 mm. The Integra CRW stereotactic system was used to perform the stereotactic biopsy.

MRI scans routinely use data from 1 to 7 days before surgery. For patients with complex and variable disease conditions, especially those with small lesions and brainstem lesions in the brain, it is strictly required to collect MRI data from 1-3 days before biopsy to ensure the MR images reflect the latest dynamic changes of the brain lesions as accurately as possible to prevent fluctuations and progression in the brain after treatment and other imaging errors.

### Stereotactic head frame placement

According to the patient's ability to cooperate, the position of the headgear is chosen. For patients who cannot cooperate and have a disturbance of consciousness, it is necessary to place the stereotactic head frame in the semi-recumbent position or the lying position. Specifically, it is necessary to pay attention to maintaining a good position and proper fixation of the frame.

The patient's degree of cooperation determines the methods of stereotactic head frame placement. The position of the frame affects the accuracy of the positioning, especially for small lesions in the brainstem or deep brain. The patient's degree of cooperation was graded as one of three levels as follows: 1) the patient was able to cooperate actively, was awake, with good general condition, and was able to sit autonomously and wear the stereotactic head frame; 2) the patient could not fully cooperate due to mild confusion or a bad body condition and required two medical staff to support their sitting position to wear the stereotactic head frame; and 3) the patient was completely unable to cooperate due to a moderate-to-severe disturbance of consciousness or poor general condition and could not maintain the sitting position, with three medical staff required to raise the patient's head (45°-60°) and maintain a semi-recumbent position for head frame placement.

### Biopsy planning

Image Fusion and Omni Sight Excel series (Integra Radionics, Inc., USA) software was used for CT-MRI fusion positioning. The incision and target were set and the coordinate values were recorded. According to the target position and image characteristics, the individualized puncture path was designed based on the following routine biopsy principles: 1) Brainstem target lesions: for lesions in the midbrain and upper pons the conventional ipsilateral frontal lobe approach was adopted. The puncture path follows the principle of parallel brainstem long axis, the incision is usually made 1-2 cm behind the coronal suture, 3-4 cm beside the midline. For the lower and lateral lesions of pons, we adopted contralateral, transfrontal, extraventricular approach, a biopsy path through the internal capsule, thalamus, midbrain, pons, and other structures, avoiding cerebellar canopy occlusion, entering the target lesions in the lower part of the pons and the brachium pontis; 2) For the target lesion in the internal capsule, thalamus, basal ganglion, and frontal lobe, the ipsilateral frontal lobe approach was adopted. The entrance point was located in front of the coronal suture and 2-3 cm from the midline; 3) For the deep target lesions in the parietal lobe, occipital lobe, and temporal lobe, the ipsilateral parietal nodule approach was routinely adopted. The entrance point was located 2-3 cm from the midline.

### Surgical and biopsy procedures

The patient usually adopted a supine position, and the CRW Stereotactic Instrument bow was installed. After skin incision, drilling the skull, and cutting the dura mater, the biopsy needle was used to puncture according to the planned system coordinates. The biopsy tissue block was taken at the 0, 4, and 8 o'clock direction. Using a Sedan side-cutting biopsy needle (Integra Radionics, Inc., USA), a columnar tissue block (1.5×1.5×10 mm) was obtained by a single spin-cutting, and a 2-mL syringe was used for gentle vacuum suction.

The sample collector must be familiar with the resistance of the brain tissue, texture and toughness of the lesion when the biopsy needle is inserted to adjust the force needed to push the biopsy needle, avoiding excessive force and rigid puncture. At the same time, when reaching the target lesion, attention should be paid to control negative suction pressure and avoid excessive suction, resulting in bleeding risk.

The maximum depth of the target was 160±5 mm. The biopsy should be taken as far as possible through the center of the lesion and the periphery. If the imaging features of the lesion were variable, 2-3 targets can be selected to increase the positive sampling rate. In this study, 56 cases had a biopsy taken from a single target and 16 cases had biopsies taken from multiple targets.

The number of samples was determined based on the bleeding condition after the puncture needle was introduced. After sampling was completed, the biopsy needle was slowly withdrawn to terminate the operation. In case of bleeding, 500∼1000 U of thrombin (dissolved in 2∼5 mL of saline) was injected into the target lesion to promote coagulation.

The blood pressure, heart rate, respiration, neurological changes, state of consciousness should be monitored during the whole sampling process. The blood pressure should be maintained at the baseline low level during the puncture process, and fluctuations in vital signs should be timely handled to avoid adverse consequences.

In biopsies in high-risk regions, such as brainstem and genu of the internal capsule, patients underwent head CT 2-4 h after surgery to monitor complications, such as bleeding. In the other regions, the head CT scan was routinely performed one day after surgery.

## Results

### Patient's cooperation with the stereotactic head frame placement

Among the 72 cases, 46 patients (63.9%) were able to cooperate actively. Ten cases (13.9%) could not fully cooperate due to mild confusion or bad body condition. Sixteen patients (22.2%) were completely unable to cooperate due to moderate-to-severe disturbance of consciousness or poor general condition.

As for anesthesia methods, 42 cases (60%) received local anesthesia and intravenous anesthesia, while 30 cases (40%) underwent general anesthesia.

### Surgery outcomes

Most patients had stable vital signs intraoperatively and operations were successful. One patient underwent a stereotactic brainstem biopsy via a contralateral approach. When the biopsy needle reached 0.5 cm above the target (brachium pontis), the patient's heart rate decreased rapidly. After the operation was suspended, the heart rate returned to normal until the biopsy was completed.

### Complications

**Figure 1:**
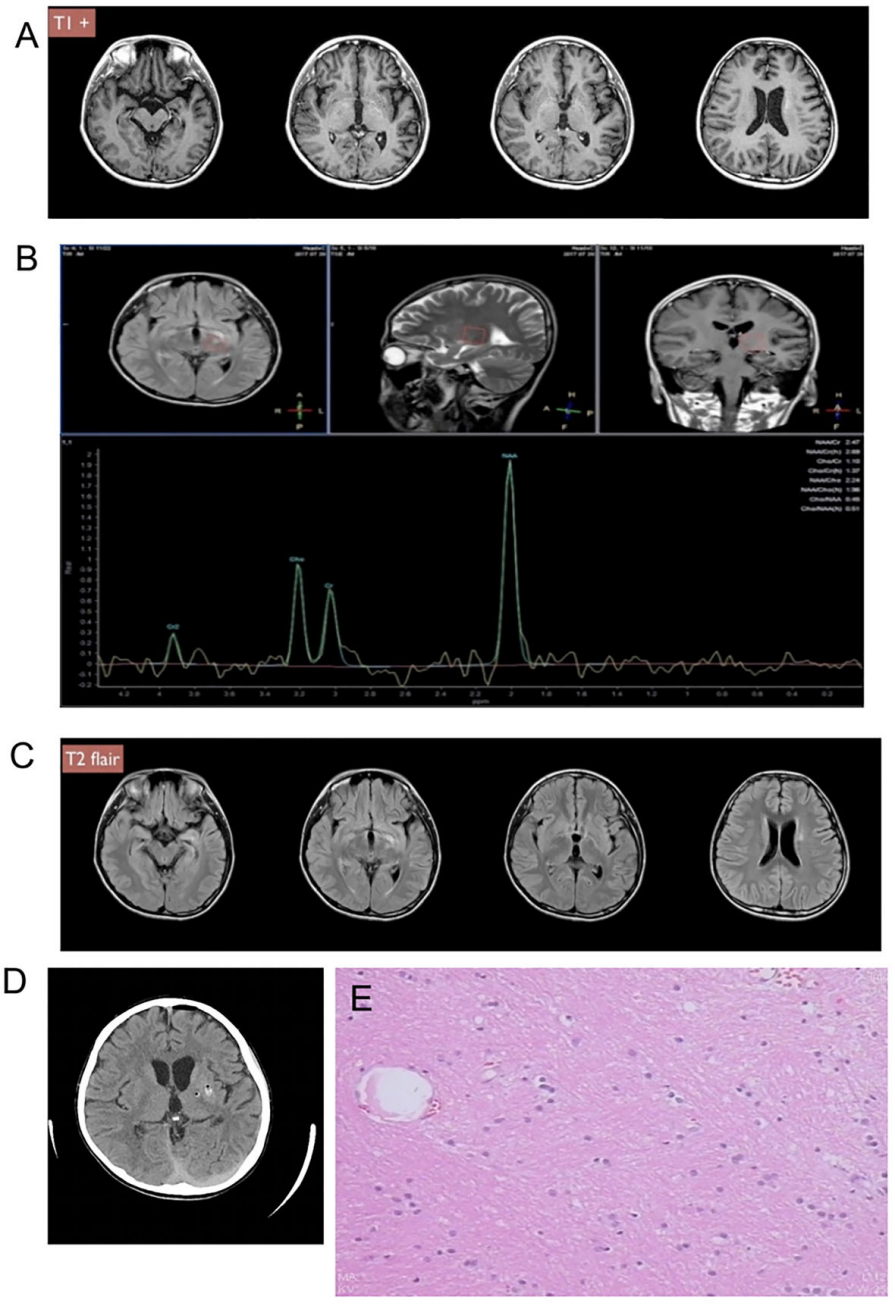
Case 1. **A**, Preoperative head T1-weighted contrast-enhanced magnetic resonance imaging (MRI) revealed left ventricle dilatation, enlarged sacral angle, and suspicious high signal on the left side of the ventricle, left basal ganglion. **B**, Magnetic resonance spectroscopy shows left basal ganglion NAA/Ch: 2.24, NAA/Cr: 2.69, Ch/Cr: 1.10. **C**, Preoperative head MRI T2 flair showed small abnormalities in the left ventricle, left midbrain, and thalamus. **D**, Computed tomography scan 2 h after surgery showed left basal ganglion micro-bleeding grade I and small areas of pneumocephalus. **E**, The pathological finding showed a single cell of a germ cell tumor.

As for post-biopsy complications, there were 3 cases of asymptomatic minor hemorrhage (4.2%), 2 cases of symptomatic minor hemorrhage (2.8%) (Figure 1D), 2 cases of massive hemorrhage (2.8%), and 2 cases of permanent neurological dysfunction (2.8%). No biopsy-related deaths occurred.

### Distribution of biopsy targets

The distribution of biopsy targets is shown in Table 1. The most common biopsy targets were deep in the lobes (43 cases, 59.7%), including frontal lobe (33 cases, 45.8%), temporal lobe (4 cases, 5.6%), parietal lobe (3 cases, 4.2%), and occipital lobe (3 cases, 4.2 %). There were 12 cases (16.7%) of brainstems, including midbrain in 8 cases (11.1%) and pons or brachium pontis in 4 cases (5.6%). Other targets included internal capsule (2 cases, 2.8%), thalamus (3 cases, 4.2%), and basal ganglion (12 cases, 16.7%).

**Table 1:** Distribution of target sites for biopsy in 72 brain lesions.

Biopsy target	Cases (%)
Brainstem	12 (16.7)
Midbrain	8 (11.1)
Pons	4 (5.6)
Internal capsule	2 (2.8)
Genu	1 (1.4)
Posterior limb	1 (1.4)
Thalamus	3 (4.2)
Basal ganglion	12 (16.7)
Deep in the lobes	43 (59.7)
Frontal lobe	33 (45.8)
Temporal lobe	4 (5.6)
Parietal lobe	3 (4.2)
Occipital lobe	3 (4.2)
Total	72 (100)

Pathological results of the 72 cases of intracerebral lesions from stereotactic biopsy are reported in Table 2. The most common pathological type was lymphoma (29.2%), followed by glioma (25.0%) and demyelination (20.8%). Of the 72 cases, 69 patients had confirmed pathological results (Table 2). The diagnosis yield of stereotactic biopsy was 95.2% (69/72).

**Table 2:** Pathological results of 72 cases of intracerebral lesions from stereotactic biopsy.

Pathological diagnosis	Cases (%)
Lymphoma	21 (29.2)
Glioma	19 (25.0)
Demyelination	15 (20.8)
Germ cell tumor	3 (4.2)
Brain parasite infection	3 (4.2)
Medulloblastoma recurrence	1 (1.4)
Metastasis	3 (1.4)
Cerebral leukemia	1 (1.4)
Tuberculous granuloma	1 (1.4)
Gliosis	1 (1.4)
Hereditary prion disease	1 (1.4)
Unknown diagnosis	3 (4.2)
Total	72 (100)

Four selected cases illustrating stereotactic biopsy strategies for lesions in the brainstem or deep in the lobes are described in the following subsections.

### Case 1

#### Left thalamus, midbrain lesions, small, and difficult lesion biopsy via internal capsule approach

A 10-year-old male patient had right hand shaking and was unable to write, accompanied by feet sliding on the right side, changes in memory/personality, and verbal reduction for 2 years, as well as diabetes insipidus, irregular fever, and asymmetric enlargement of the penis and testicles for one year.

The patient had a long disease course and underwent misdiagnosis and mistreatment for 2 years. He underwent a variety of blood tests, imaging examinations, and only endocrine abnormalities were identified (low specific gravity urine, abnormal sex hormones). MRI (Figure 1A) and MR spectroscopy (Figure 1B) showed a small abnormal signal in the left basal ganglion, and imaging diagnosis considered demyelinating lesions. MRI showed that the lesions were small and could not be diagnosed without a biopsy. Two small abnormal signals of T2 flair in the left thalamus and midbrain junction were selected as biopsy targets (Figure 1C).

The biopsy path passed through the genu of the internal capsule. Two hours after the operation, CT showed a small area of pneumocephalus (Figure 1D). Although there was no obvious bleeding, a right limb hemiplegia occurred 3 days after surgery, perhaps due to the local edema of the genu of the internal capsule after the puncture injury, resulting in dysfunction. After treatment for hemiplegia, the patient recovered to preoperative status 1 week after surgery. A pathological diagnosis of a germ cell tumor was made (Figure 1E).

### Case 2

#### Biopsy of highly enhanced lesion in the genu of internal capsule

**Figure 2:**
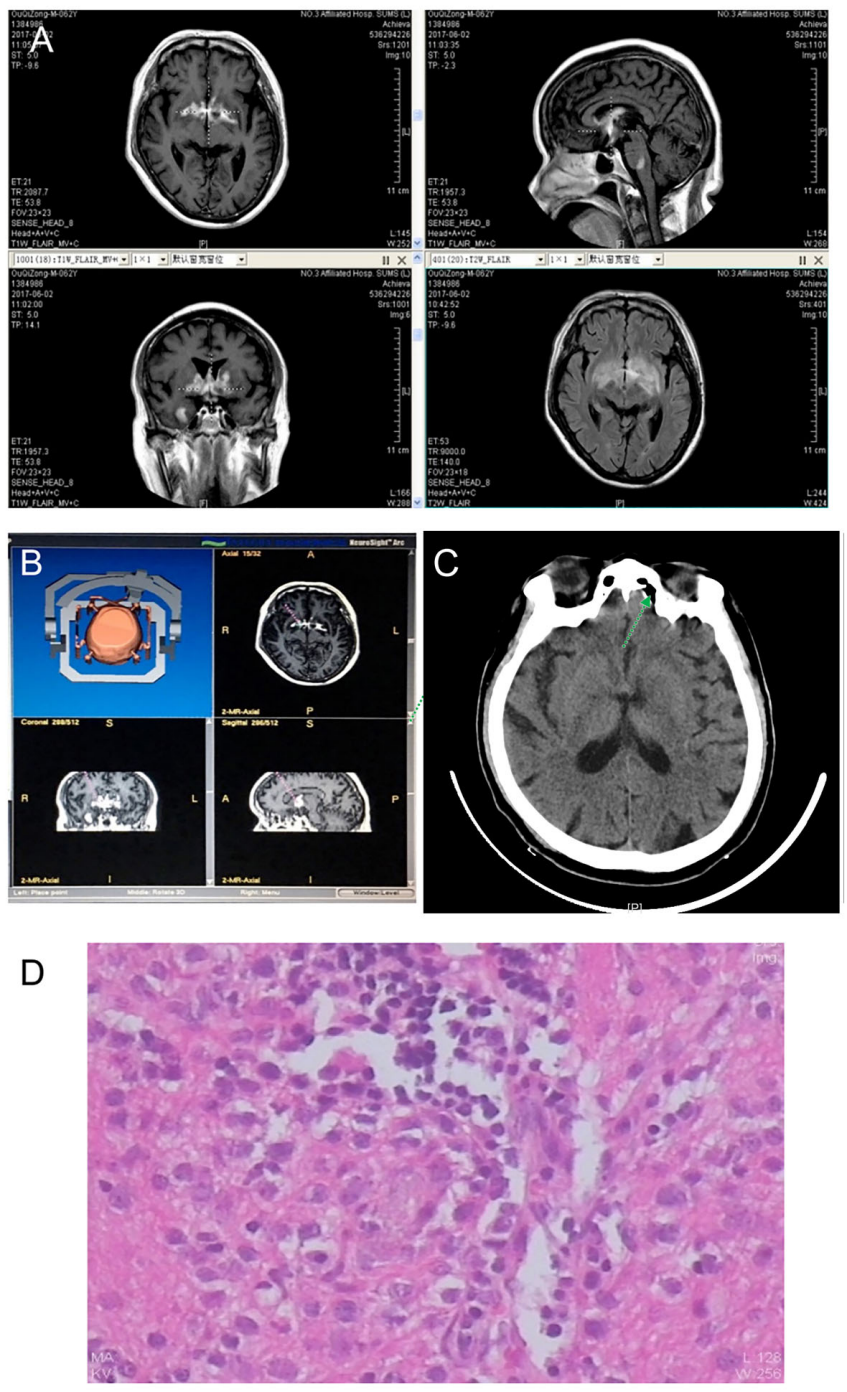
Case 2. **A**, T1-weighted contrast-enhanced magnetic resonance imaging (MRI) showed a bilateral internal capsule, corpus callosum, and a high-intensity lesion in pons-medulla junction. MRI T2 flair showed an abnormally high signal. **B**, Three-dimensional surgical plan - right internal capsule target. **C**, Computed tomography 1 day after surgery; the arrow shows small areas of pneumocephalus in the genu of the right internal capsule. **D**, Pathological diagnosis of diffuse large B-cell lymphoma.

A 62-year-old male patient complained of memory loss for one month. MRI scan found lesions in the bilateral internal capsule, basal ganglion, optic chiasm, pituitary stalk, and pons (Figure 2A). Imaging diagnosis was lymphoma.

The right genu of internal capsule enhanced lesions was selected as biopsy targets (Figure 2B). There was no new neurological dysfunction found after surgery (Figure 2C). A pathological diagnosis of diffuse large B-cell lymphoma was made (Figure 2D).

### Case 3

#### Transfrontal, extraventricular approach to micro-lesions biopsy in the pons-medulla junction

A 39-year-old female patient complained of repeated headaches for 2 years, aggravated unclear vision, binaural hearing hypersensitivity, left facial numbness, and unstable walking. Physical examination showed the left eye was involuntarily closed, limited abduction of the right eye, inability of abduction and limited adduction of the left eye, the left side showed hypoalgesia and hyperacusis, muscle strength of the left lower extremity was grade 5, the quadriplegia reflex was (+++), and the bilateral pyramidal sign was (+).

PET-CT showed no signs of hypermetabolic malignancy, and the pons-medullary junction was slightly thickened. No abnormal increase in metabolism, and the imaging diagnosis considered inflammatory lesions.

The patient was diagnosed with brainstem encephalitis in other hospitals, and the symptoms were relieved after hormone treatment but then aggravated again.

**Figure 3:**
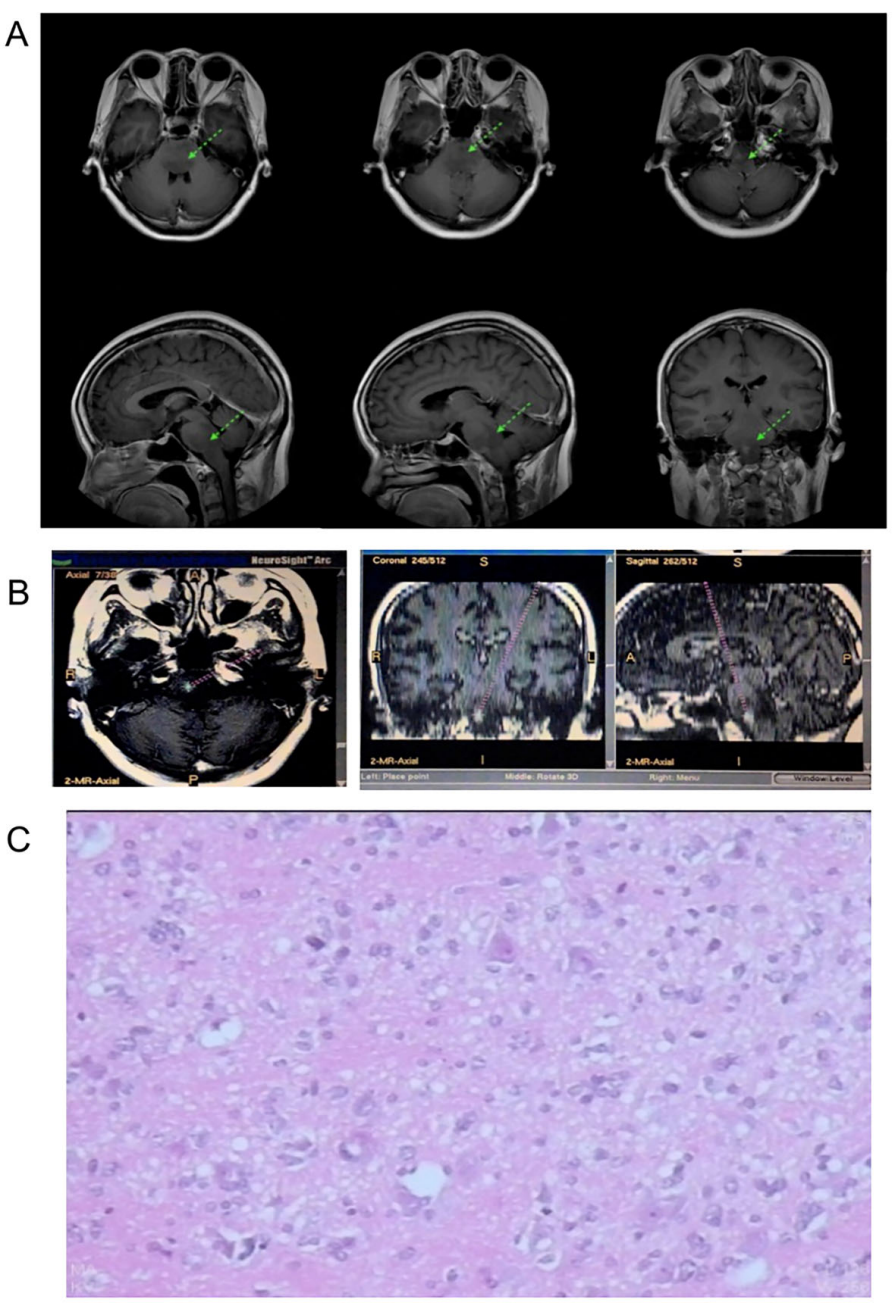
Case 3. **A**, T1-weighted contrast-enhanced magnetic resonance imaging showed pons, pons-medulla abnormally low signal, and no obvious enhancement. **B**, Surgical planning for micro-targets across the midline and pons-medulla junction. **C**, Pathological diagnosis of WHO Grade III gliomas.

Brain MRI T1 enhancement showed lesions in the midbrain, pons, and medullary diffuse low-signal lesions, without significant enhancement (Figure 3A). A transfrontal, extraventricular approach to micro-lesions biopsy in the pons-medulla junction was adopted (Figure 3B). Pathological diagnosis was World Health Organization (WHO) Grade III gliomas (Figure 3C).

### Case 4

#### Transfrontal, extraventricular approach to atypical lesions biopsy in the brachium pontis

A 24-year-old male patient had coughing when drinking water and difficulty swallowing for 10 months, aggravated for 1 month. MRI scan showed pons swelling, slightly high pons-medulla signal, indicating non-tumor lesions (brainstem encephalitis). Hormone and gamma treatment relieved the symptoms, but aggravation returned. Physical examination showed dysarthria, hoarseness, shallowing of the left nasolabial fold, right deviation of the tongue, hyperreflexia of the extremities, and bilateral Babinski sign (+). MRI plain scan + enhancement + diffusion-weighted imaging+ magnetic resonance spectroscopy + magnetic resonance angiography + magnetic resonance venography only showed a slightly higher signal in the T2 flair sequence. PET-CT showed no highly metabolic lesions in the whole body but limited high metabolism in the brainstem.

**Figure 4:**
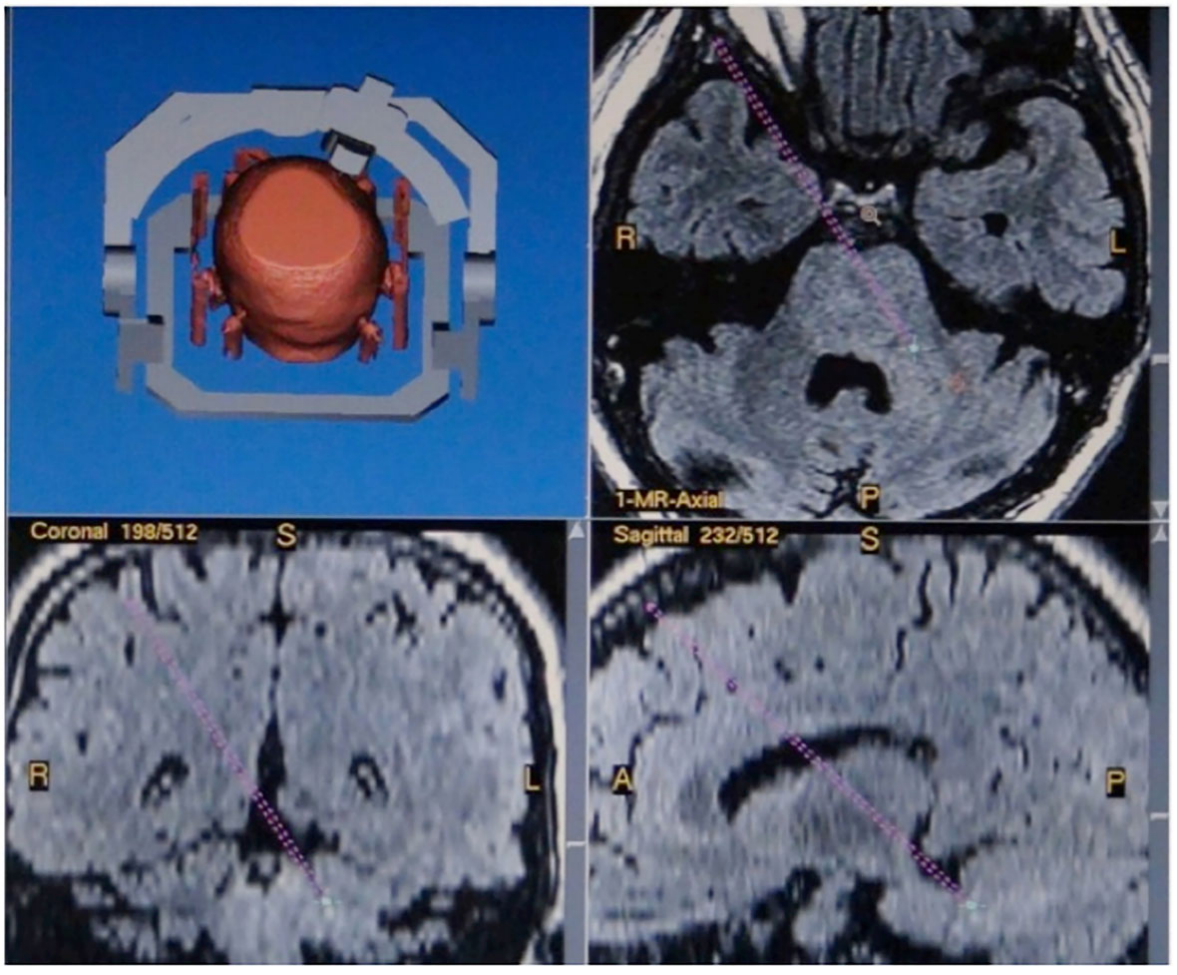
Case 4. Three-dimensional surgical plan: the extraventricular approach to suspicious biopsy targets on the left side of the brachium pontis.

A transfrontal, extraventricular approach to atypical lesions biopsy in brachium pontis was performed (Figure 4). The pathological diagnosis was WHO Grade II diffuse astrocytoma.

## Discussion

Accumulating evidence has suggested that stereotactic biopsy of the brainstem and deep brain lesions is accurate, safe, and has a low incidence of complications (5,7,10,17-19). At present, however, the brain lesions that can be biopsied based on the imaging characteristics evaluated by neurologists and neurosurgeons are still limited. For example, because of the need to contrast-enhance lesions in MRI, a biopsy is often abandoned due to the high risk of bleeding. For some important areas (such as the internal capsule area) and high-risk areas (such as the pons-medulla junction and the medulla), biopsy cases are extremely rare. Callovini et al. (20) analyzed a series of 421 stereotactic brain biopsy cases treated between January 2002 and June 2017 in three major neurosurgical institutes in Rome and found that there was a significant reduction in stereotactic brain biopsy procedures in lobar lesions, while those performed on the basal ganglia and of multiple masses increased. However, in our 72 cases of biopsy, deep lobar lesions accounted for 56.9%, mainly diffuse, deep, intolerant to radical resection, and atypical imaging lesions. A stereotactic biopsy was required to obtain tissue for pathological diagnosis. In the research by Callovini et al. (20), intraoperative frozen sections were performed in 78% of the biopsies. It has been shown that intraoperative frozen sections biopsy did not affect the diagnostic yield of stereotactic brain biopsy (21). We do not routinely perform intraoperative frozen section biopsy during a stereotactic brain biopsy. Nevertheless, the diagnostic yield of stereotactic brain biopsy reached 95.2% in this study, which is similar to that of biopsy verified by intraoperative frozen pathology (6).

In this study, we focused mainly on difficult lesions in the brainstem and deep brain, and important high-risk areas, such as the brainstem, internal capsule, thalamus, and basal ganglia, accounted for 40.4% of all cases. This study included 2 cases of the genu of internal capsule highlighted lesions and 1 case of pons-medulla junction small lesion biopsy. After internal capsule and brainstem biopsy, transient neurological dysfunction may occur but without permanent neurological dysfunction. These results showed that lesions in high-risk areas can also be safely biopsied, which is worthy of further investigation.

The approach to brain lobe lesions can be selected according to the location of the target. For lesions in the frontal lobe, sellar region, and basal ganglia, the access hole can be drilled at 3 cm away from the ipsilateral coronal suture and the sagittal suture. For lesions in the temporal lobe, parietal lobe, occipital lobe, and pineal region, the access hole can be drilled posterior to the parietal tuber. In the brain lobes and basal ganglia biopsies, special attention should be paid to vascular lesions, high signal intensity lesions, and fast-growing malignant lesions. It has been shown that deep-seated primary central nervous system lymphomas have a high risk of biopsy-related morbidity and mortality, mainly due to a high risk of post-biopsy hemorrhage (22). Supporting this notion, among the 72 cases in this study, the 2 cases with postoperative massive hemorrhage were diffuse large B-cell lymphomas. The high risk of post-biopsy hemorrhage may not be related to the length of the puncture path, but to the pathological type of primary central lymphoma. Studies also recommend that postoperative CT be performed in patients with suspected primary central nervous system lymphoma (22). In this study, all cases routinely received postoperative CT to rule out post-biopsy intracranial hemorrhage.

As a dense area of the nerve conduction bundle in the brain, the function of the internal capsule region is very important. There is no report of biopsy of internal capsule targets or biopsy via the internal capsule approach, but this study suggested that those are feasible and safe, providing a reference for future research. Nevertheless, when designing a biopsy puncture path strategy, the inner capsule area should be avoided as much as possible to prevent the potential risk of neurological dysfunction.

In this study, there were 2 cases of successful biopsy in the internal capsule area (2.8%). The internal capsule lesions can be biopsied after careful evaluation and design of the biopsy path. Case 2 of this study was a high signal enhanced lesion biopsy of the genu of the internal capsule, which was diagnosed as diffuse large B-cell lymphoma by biopsy and no new neurological dysfunction was found postoperatively. The other case was an enhanced lesion biopsy of the posterior limb of the internal capsule. The intraoperative biopsy was performed 8 times. The postoperative CT scan showed that the amount of bleeding was about 0.6 mL. The biopsy was strictly confined to the lesioned tissue, without causing significant bleeding and no new dysfunction after surgery, suggesting that the biopsy procedure only damages the lesion tissue and has little effect on normal tissue. After rehabilitation, the patient's left upper limbs were less flexible, and the muscle strength reached 4^+^. These results suggested that the internal capsule was not a restricted area for biopsy.

Amundson et al. (23) and Pereira et al. (24) reported that in the contralateral frontal, midline-crossing approach for brain biopsy, the biopsy path can be through the lateral ventricle, internal capsule, thalamus, via the midbrain and aqueduct, and pontine, then reaching the target biopsy site. Although the length of this biopsy path is long, it can effectively avoid the obstruction of the tentorium. With well-designed preoperative planning, no serious complications occurred. As of December 2019, 4 patients had received stereotactic brain biopsy via contralateral frontal, midline-crossing approach in our hospital. All operations were successful and there were no novel neurological dysfunctions. Therefore, these results suggested that this approach is an alternative strategy for the biopsy of the lateral brainstem lesions.

Case 1 was a tiny lesion biopsy of the left thalamus and midbrain junction. The three-dimensional surgical plan showed that the genu of the internal capsule could be avoided in the puncture route. Two hours after the biopsy, the head CT showed cranial pneumatosis (small air bubbles) in the genu of the internal capsule, microbleeding in the basal ganglia, and no new neurological dysfunction in the nervous system examination. The patient developed hemiplegia of the contralateral limbs 3 days after surgery, and the muscle strength was grade 2. After conservative treatment, the limb muscle strength returned to the preoperative state at 7 days after surgery and the patient was discharged. Therefore, this result suggested that the genu of the internal capsule can be used as a puncture route under a rigorous and thorough three-dimensional surgical plan design. Patients may develop transient neurological dysfunction, but generally do not have permanent neurological dysfunction. For the internal capsule area as a puncture route, a rigorous risk-benefit assessment should be performed before surgery. If sufficient biopsy specimens have been obtained for diagnosis, the number of punctures should be reduced as much as possible to decrease the risk of local injury and small bleeding, reducing the incidence of complications, such as neurological dysfunction.

It has been suggested that in the biopsy for lesions in the midbrain and upper pons, an ipsilateral frontal approach is recommended, and the drilling hole can be at 2 cm behind the coronal suture and 3 to 4 cm apart from the midline, keeping the puncture path parallel to the long axis of the brainstem (conducting bundle) (7,17,25). As for the lower brainstem region, such as the lower pons and the brachium pontis, the posterior cranial fossa (cerebellar) approach is recommended, and the drilling hole can be at 4 cm below the occipital trochanter and 3 cm apart from the midline, which is the shortest path (7,17,25).

Previous large studies have shown that brainstem biopsy has high safety, and the incidence of permanent disability and mortality ranges from 0.7 to 1.5% (25-27). Tobin et al. (27) report that, among 137 cases of brainstem or cerebellar parenchymal biopsy, all 3 deaths occurred when using the cerebellar approach for biopsy. Two patients died at the time of biopsy and 1 died of complications. When using the cerebellar approach, due to the obstruction of the stereotactic frame, the steps of skin incision, skull drilling, and the dural incision cannot be performed under direct vision. Once puncture bleeding occurs at the skull, dura mater, and/or cerebellar cortex, the space of the posterior cranial fossa is of poor compensatory ability due to its small volume, easily causing cerebellar tonsillar hernia and life-threatening condition. Therefore, it is necessary to increase the risk awareness for cerebellar biopsy.

In this study, a contralateral, transfrontal, extraventricular approach was used as an alternative for the cerebellar approach to reduce the risk of bleeding when performing a blind puncture into the skull. At present, the mainstream opinion is that the ipsilateral approach to brain lesions should be routinely used for biopsy. The contralateral, transverse approach to the lesion is regarded as an unreasonable and unsafe surgical path, especially for low brainstem lesions. In this long surgical path, the needle needs to puncture a series of the most important functional regions, including the inner capsule, the midbrain, and the pons, which is considered a surgical restriction in most functional neurosurgery, thus counteracting the conventional surgical concept of craniocerebral biopsy. Thus far, only 7 cases of the contralateral extraventricular approach to stereotactic brainstem biopsy have been reported in the USA (23) and UK (24).

In this study, 2 patients received contralateral, extraventricular approach to low brainstem biopsy, one case of local anesthesia and one case of general anesthesia, and no new postoperative neurological dysfunction was found. Case 3 was a small enhanced lesion in the right pons-medulla junction. It is difficult to obtain sufficient lesioned tissue for biopsy through the ipsilateral cerebellar approach. The three-dimensional surgical planning system showed that the puncture path needed to penetrate the lateral ventricle wall, unable to avoid the cisterna ambiens, leading to a high risk of bleeding. We thus designed a biopsy path via the left frontal lobe, contralateral, extraventricular approach to the pons-medulla junction, and biopsy was successfully carried out. In Case 4, the target lesion was located in the left brachium pontis, and a biopsy path via the right frontal lobe, the extraventricular approach was adopted, passing through the lateral ventricle, internal capsule, thalamus, the midbrain, and the pons, to the target lesion in the pons-medulla junction, achieving accurate and safe biopsy. Compared to previous studies (23,24), the biopsy targets were more blurred and smaller in this study. These results suggested that under the premise of a prudent and rigorously designed three-dimensional surgical planning strategy, the contralateral, extraventricular approach is an alternative to the ipsilateral cerebellar approach to brainstem biopsy, which can avoid bleeding in the skull, dura mater, and cerebellum cortex caused by the cerebellum and non-direct vision.

One of the limitations of this study was the relatively small sample size. The findings of this study should be validated in a large study.

In summary, the CT-MRI-guided stereotactic biopsy of lesions in the brainstem or deep brain has the advantages of high safety, accurate diagnosis, and low incidence of complications. It plays a crucial role in the diagnosis of atypical, microscopic, diffuse, multiple, and refractory lesions. In the extraventricular approach to low-brainstem biopsy, internal capsule biopsy, and genu of internal capsule biopsy, a well-designed individualized surgical strategy can reduce neurological dysfunction and achieve a safe biopsy.

## References

[1] Youmans JR, Winn HR (2011). Youmans neurological surgery.

[B02] Mabray MC, Barajas RF, Cha S, Cha S (2015). Modern brain tumor imaging. Brain tumor Res Treat.

[B03] Hankinson TC, Campagna EJ, Foreman NK, Handler MH (2011). Interpretation of magnetic resonance images in diffuse intrinsic pontine glioma: a survey of pediatric neurosurgeons. J Neurosurg Pediatr.

[4] Zimmerman RA (1996). Neuroimaging of primary brainstem gliomas: diagnosis and course. Pediatr Neurosurg.

[5] Bai HX, Zou Y, Lee AM, Lancaster E, Yang L (2015). Diagnostic value and safety of brain biopsy in patients with cryptogenic neurological disease: a systematic review and meta-analysis of 831 cases. Neurosurgery.

[6] Dammers R, Schouten JW, Haitsma IK, Vincent AJPE, Kros JM, Dirven CMF (2010). Towards improving the safety and diagnostic yield of stereotactic biopsy in a single centre. Acta Neurochir (Wien).

[7] Rachinger W, Grau S, Holtmannspötter M, Herms J, Tonn JC, Kreth FW (2009). Serial stereotactic biopsy of brainstem lesions in adults improves diagnostic accuracy compared with MRI only. J Neurol Neurosurg Psychiatry.

[8] Yang C, Wei X, Niu C, Fu X, Qian R, Yehan W (2011). Diagnostic value of stereotactic biopsy in intracranial lesions without enhancement effect [in Chinese]. Chinese J Minim Invasive Neurosurg.

[9] Zhao S, Zhang J, Chang H, Sun Y, Liu J, Yin F (2015). Factors affecting diagnostic yield of stereotactic biopsy in non-enhancement brain lesions [in Chinese]. Chinese J Neurosurg Dis Res.

[10] Gessler F, Baumgarten P, Bernstock JD, Harter P, Lescher S, Senft C (2017). Assessment of molecular markers demonstrates concordance between samples acquired via stereotactic biopsy and open craniotomy in both anaplastic astrocytomas and glioblastomas. J Neurooncol.

[11] Nishihara M, Kohmura E, Takeda N, Harada T, Kidoguchi K, Tatsumi S (2014). Diagnostic yield and morbidity by neuronavigation-guided frameless stereotactic biopsy using magnetic resonance imaging and by frame-based computed tomography-guided stereotactic biopsy. Surg Neurol Int.

[12] Giannetti AV, Alvarenga AYH, de Lima TOL, Pedrosa HA-SR, Souweidane MM (2015). Neuroendoscopic biopsy of brain lesions: accuracy and complications. J Neurosurg.

[13] Can SM, Turkmenoglu ON, Tanik C, Uysal E, Ozoner B, Kaldirimoglu SA (2016). Computerized tomography guided stereotactic biopsy of intracranial lesions: report of consecutive 500 cases. Turk Neurosurg.

[14] Larson PS, Starr PA, Bates G, Tansey L, Richardson RM, Martin AJ (2012). An optimized system for interventional magnetic resonance imaging-guided stereotactic surgery: preliminary evaluation of targeting accuracy. Neurosurgery.

[15] Qin F, Huang Z, Cai M, Xu X, Lu T, Dong Q (2018). Stereotactic biopsy in the accurate diagnosis of lesions in the brain stem and deep brain [in Chinese]. Zhonghua Yi Xue Za Zhi.

[16] Huang ZC, Dong Q, Song EP, Chen ZJ, Zhang JH, Hou B (2020). Germinomas of the basal ganglia and thalamus: four case reports. World J Clin Cases.

[17] Kickingereder P, Willeit P, Simon T, Ruge MI (2013). Diagnostic value and safety of stereotactic biopsy for brainstem tumors: a systematic review and meta-analysis of 1480 cases. Neurosurgery.

[B18] Schumacher M, Schulte-Mönting J, Stoeter P, Warmuth-Metz M, Solymosi L (2007). Magnetic resonance imaging compared with biopsy in the diagnosis of brainstem diseases of childhood: a multicenter review. J Neurosurg.

[19] Iijima K, Hirato M, Miyagishima T, Horiguchi K, Sugawara K, Hirato J (2015). Microrecording and image-guided stereotactic biopsy of deep-seated brain tumors. J Neurosurg.

[20] Callovini GM, Telera S, Sherkat S, Sperduti I, Callovini T, Carapella CM (2018). How is stereotactic brain biopsy evolving? A multicentric analysis of a series of 421 cases treated in Rome over the last sixteen years. Clin Neurol Neurosurg.

[21] Tsermoulas G, Mukerji N, Borah AJ, Mitchell P, Ross N (2013). Factors affecting diagnostic yield in needle biopsy for brain lesions. Br J Neurosurg.

[22] Callovini GM, Sherkat S, Sperduti I, Crispo F, Raus L, Gazzeri R (2021). Hemorrhagic attitude in frameless and frame-based stereotactic biopsy for deep-seated primary central nervous system lymphomas in immunocompetent patients: a multicentric analysis of the last twenty years. World Neurosurg.

[23] Amundson EW, McGirt MJ, Olivi A (2005). A contralateral, transfrontal, extraventricular approach to stereotactic brainstem biopsy procedures. J Neurosurg.

[24] Pereira EAC, Jegan T, Green AL, Aziz TZ (2008). Awake stereotactic brainstem biopsy via a contralateral, transfrontal, transventricular approach. Br J Neurosurg.

[25] Hamisch C, Kickingereder P, Fischer M, Simon T, Ruge MI (2017). Update on the diagnostic value and safety of stereotactic biopsy for pediatric brainstem tumors: a systematic review and meta-analysis of 735 cases. J Neurosurg Pediatr.

[26] Dellaretti M, Reyns N, Touzet G, Dubois F, Gusmão S, Pereira JLB (2012). Stereotactic biopsy for brainstem tumors: Comparison of transcerebellar with transfrontal approach. Stereotact Funct Neurosurg.

[27] Tobin WO, Meyer FB, Keegan BM (2015). Diagnostic yield and safety of cerebellar and brainstem parenchymal biopsy. World Neurosurg.

